# The fetoneonatology fellowship: a unified subspecialty for the fetal–neonatal continuum

**DOI:** 10.1038/s41372-026-02792-w

**Published:** 2026-07-01

**Authors:** Thomas Hegyi

**Affiliations:** Department of Pediatrics, Division of Neonatology, Robert Wood Johnson Medical School, Rutgers University, New Brunswick, NJ USA

**Keywords:** Health occupations, Scientific community

## Abstract

Advances in fetal diagnosis and therapy have created a need for clinicians with expertise spanning prenatal assessment and neonatal care. Current maternal fetal medicine and neonatology training pathways remain largely separate, leaving gaps in knowledge of placental pathology, fetal surveillance, antenatal interventions, and their neonatal consequences. We propose a fetoneonatology fellowship for graduates of neonatal and potentially obstetric residency programs at academic centers with maternal fetal medicine services, Level IV neonatal intensive care units, and placental pathology expertise. The three-year curriculum would include one year of fetal medicine and placental biology and two years of neonatal intensive care training integrated through a Fetal Neonatal Bridge Curriculum. An optional advanced track would emphasize continuity care, research, and artificial intelligence-supported practice. This model aims to improve prenatal neonatal correlation, trainee preparedness, and family communication, and could evolve from a super fellowship into an accredited subspecialty.

## A patient at the intersection of everything

*She is 27 weeks and 4 days of gestation. Her mother has chronic hypertension on labetalol, gestational diabetes requiring insulin, and a prior preterm birth at 31 weeks. Doppler studies today show absent end-diastolic flow in the umbilical artery and a cerebroplacental ratio below the 5th centile. The estimated fetal weight is 610 grams, below the 3rd centile. Betamethasone was administered 4 days ago; magnesium sulfate is being administered for neuroprotection. Delivery by cesarean is planned for tomorrow morning*.

Five specialists are involved in her care tonight. The maternal-fetal medicine (MFM) physician made the delivery decision based on Doppler criteria but has not been trained in the specific pattern of neonatal morbidity that absent end-diastolic flow predicts, including polycythemia, hypoglycemia, elevated risk of necrotizing enterocolitis, and impaired postnatal brain growth, which will appear on head ultrasound at 1 week of age [1]. The neonatologist knows these neonatal sequelae but has not been trained in the placental pathology report arriving in 72 h, or in what the maternal vascular malperfusion pattern documented there reveals about the intrauterine inflammatory milieu to which this infant’s developing brain has been exposed for weeks [2, 3]. The placenta, which is the most consequential organ this infant will ever have, belongs formally to neither.

This is not a failure of individual physicians. It is a failure of the structure within which they were trained. It is precisely the structure that the fetoneonatology fellowship is designed to replace.

## The delivery room divide: an organizational artifact with biological costs

Modern neonatology and maternal-fetal medicine developed as parallel disciplines, sharing a single patient. The high-risk fetus and the high-risk neonate are, by definition, the same individual, but they built separate training pathways, separate certifying examinations, and separate intellectual cultures. This separation was historically defensible. The investigative tools of each field were largely distinct, and the two worlds touched at the delivery room door and then separated again.

That justification has expired. Three decades of research have established beyond reasonable dispute that the fetal and neonatal periods are not sequential chapters. They are a single continuous physiologic narrative in which intrauterine conditions directly encode neonatal vulnerability, neonatal management alters developmental trajectories established before birth, and the boundaries between obstetric and neonatal decision-making are, in biological terms, entirely artificial.

The evidence is disease-specific. Necrotizing enterocolitis was understood for decades as a postnatal disease of prematurity and formula feeding. It is now recognized that chorioamnionitis, an intrauterine inflammatory process, fundamentally alters the developing intestinal microbiome and mucosal immune architecture before delivery [4], and that placental insufficiency impairs mesenteric blood flow in utero, establishing vulnerability that the postnatal environment then exploits. The MFM physician’s timing-of-delivery decision in the growth-restricted fetus is, in this light, a decision about NEC risk, yet no formal mechanism ensures that this understanding is operative in either physician’s clinical reasoning.

Intraventricular hemorrhage (IVH) follows the same logic. The germinal matrix’s vulnerability to hemorrhage is shaped by gestational age, intrauterine growth restriction, the maternal inflammatory milieu, and a history of corticosteroid exposure, all of which are determined before the neonatologist is involved [5, 6]. Maternal vascular malperfusion on placental pathology is an independent predictor of IVH, a connection that spans the delivery room divide and that neither specialty, trained in isolation, is equipped to translate into practice [7]. Bronchopulmonary dysplasia is increasingly understood as a disease of arrested lung development whose inflammatory origins are intrauterine, modulated by placental function and chorioamnionitis [8].

Bodenheimer identified care fragmentation as a structural generator of harm, not a mere inconvenience [9]. In the fetal-neonatal context, that harm is specific, as critical information about intrauterine exposures fails to reach clinicians who could act on it, and the biological unity of the patient is invisible to the organizational structure meant to serve that patient.

## A science without a specialty

The Developmental Origins of Health and Disease (DOHaD) framework, articulated by Barker and extended by decades of subsequent research, establishes fetal growth restriction and preterm birth as among the most powerful early-life determinants of adult cardiometabolic health that include susceptibility to hypertension, type 2 diabetes, obesity, and cardiovascular disease through epigenetic mechanisms operating during sensitive developmental windows [10, 11]. This framework is one of the most extensively replicated bodies of evidence in developmental biology. What it has not generated is a clinical specialty trained to apply it at the bedside. The neonatologist managing a growth-restricted 28-week infant is managing a patient whose adult health trajectory is being shaped, in part, by NICU decisions about nutrition, stress hormone exposure, and sensory experience. No current training pathway prepares neonatologists to reason explicitly within the DOHaD framework as a clinical tool. The fetoneonatologist would.

The history of antenatal corticosteroids makes the same point historically. Liggins and Howie’s landmark 1972 trial demonstrating that antenatal betamethasone reduces neonatal respiratory distress syndrome and death [12] was possible only because its authors thought across the delivery room divide. The therapy that was obstetric in its administration and neonatal in its effect was a fetoneonatologic intervention before the discipline existed. That it took more than two decades for antenatal corticosteroids to become standard of care in the United States has been attributed, in part, to the organizational separation between the obstetric and neonatal communities, who needed to champion it together [13]. Fetoneonatology is, among other things, an institutional structure designed to prevent that kind of delay from happening again.

## Core knowledge domains

Fetoneonatology is not maternal-fetal medicine plus neonatology. It is a distinct discipline organized around the fetal-neonatal patient, beginning at placentation and extending through neonatal hospitalization and early post-discharge follow-up. Its knowledge base encompasses five integrated domains, with specific areas listed below.

- *Placental biology and pathology*. Trophoblast invasion and spiral artery remodeling; the pathogenesis of preeclampsia, fetal growth restriction, and placental abruption; Amsterdam Placental Workshop Group lesion classification; interpretation of placental pathology reports in the context of specific neonatal outcomes; umbilical and middle cerebral artery Doppler hemodynamics as expressions of villous pathology; and placental epigenomics. A fetoneonatologist arriving at a delivery has reviewed the Doppler trajectory, holds a pre-formed model of the likely placental pathology, and enters the NICU with a specific, evidence-based prediction of neonatal vulnerability, not a blank clinical slate.
- *Fetal physiology and antenatal surveillance*. Fetal hemoglobin kinetics and oxygen dissociation; the brain-sparing redistribution response to hypoxia; fetal sleep-wake cycling and neuroendocrine stress responses; biophysical profile interpretation and its correlations with fetal acid-base status; the full Doppler spectrum (umbilical artery, middle cerebral artery, ductus venosus, uterine artery); and fetal scalp pH and lactate at delivery. The fetoneonatologist would be formally credentialed in fetal Doppler ultrasonography, closing a competency gap that currently leaves the neonatologist dependent entirely on secondhand surveillance reports.
- *Preterm birth biology and antenatal therapies*. The infectious, inflammatory, and cervical pathways to preterm birth; fetal inflammatory response syndrome (FIRS) and its neonatal organ consequences [14]; the timing-of-delivery calculus in the growth-restricted fetus; and the full pharmacologic arc of every major antenatal intervention: corticosteroids (lung maturation, HPA axis programming, interactions with maternal diabetes), magnesium sulfate (neuroprotective mechanism, neonatal hypotonia), indomethacin (ductal constriction, oligohydramnios), insulin and oral hypoglycemics (fetal hyperinsulinism, neonatal hypoglycemia), antihypertensives (neonatal bradycardia, hypoglycemia), and antibiotics for PPROM (microbiome colonization, neonatal antibiotic stewardship). For every antenatal drug currently administered by one physician and managed in the neonate by another, the fetoneonatologist holds both ends of the chain.
- *Neonatal intensive care medicine*. The full neonatology curriculum, identical in scope and duration to current fellowship training, taught within a framework that explicitly connects each major neonatal condition to its intrauterine determinants: RDS and BPD (antenatal corticosteroid-to-surfactant continuum; chorioamnionitis-driven alveolar arrest); IVH and white matter injury (germinal matrix biology; maternal vascular malperfusion as predictor); NEC (fetal mesenteric vulnerability; intrauterine microbiome programming); neonatal hypoglycemia (hyperinsulinism as sequela of maternal diabetes); PDA (ductal biology; indomethacin exposure effects); early-onset sepsis (maternal flora, chorioamnionitis, FIRS); and neonatal nutrition (fetal accretion physiology; DOHaD-aware feeding design). Transition physiology and delivery room resuscitation, including the molecular regulation of lung fluid clearance, the physiology of pulmonary vascular transition, NRP-based resuscitation including PEEP and oxygen titration, and periviability ethics, are taught with explicit reference to the intrauterine history that shapes each infant’s delivery room trajectory.
- *Neurodevelopment and the DOHaD framework in clinical practice*. Normal fetal brain development by gestational age; neurologic consequences of preterm birth across the periviable-to-late-preterm spectrum; aEEG and brain MRI interpretation; neuroprotective NICU practices (NIDCAP, family-integrated care); HIE and therapeutic hypothermia; and neurodevelopmental follow-up with standardized assessment tools. Uniquely, this domain includes formal training in applying the DOHaD evidence clinically, communicating long-term cardiometabolic risk to families of growth-restricted and preterm infants, and designing NICU care protocols, particularly around brain development, glucocorticoid exposure and postnatal nutrition, that explicitly minimize adverse developmental programming.

## A three-phase fellowship training framework

Fetoneonatology is envisioned as a subspecialty fellowship analogous to established super-fellowships in other disciplines. It is comparable, for example, to clinical cardiac electrophysiology or advanced imaging within cardiology, or to hemodynamics and point-of-care ultrasound fellowships in neonatology. Like those programs, it supplements broad specialty training with deep, integrated expertise at a clinical interface that currently lacks a formally trained practitioner. The fellowship can be pursued via two pathways: a core 3-year track (Phases 1 and 2), suitable for initiation as a 1- to 3-year super-fellowship without requiring new Accreditation Council for Graduate Medical Education (ACGME) subspecialty accreditation; or the full 5-year track incorporating the advanced Phase 3.

## Eligibility and entry requirements

The primary entry pathway is completion of a 3-year pediatric residency (PGY-1 through PGY-3). This aligns with the traditional neonatology fellowship entry point and ensures foundational competency in neonatal and pediatric medicine. In recognition of the intrinsically interdisciplinary nature of the specialty, however, the fellowship is also open to graduates of obstetrics and gynecology, maternal-fetal medicine, family medicine, and general surgery residencies, with a structured supplementary curriculum of 3 to 6 months covering neonatal physiology, newborn examination, and NICU procedures. This broadened eligibility mirrors the collaborative intellectual heritage of the discipline and may substantially expand the applicant pool at a time when interest in traditional neonatology fellowships is under pressure.

It is worth noting that the American Board of Pediatrics (ABP) has recently moved toward a 2-year clinical fellowship model in neonatology. The fetoneonatology fellowship, by contrast, proposes a 3-year minimum for the core track, with the expectation that this added year, dedicated entirely to fetal medicine and placental biology, provides genuinely distinct competency not duplicable within a compressed neonatology curriculum. The contrast is intentional and reflects a philosophical commitment to depth over efficiency in training a new kind of integrative specialist.

## Phase 1: fetal medicine and placental biology (year 1)

Year 1 is embedded in a high-volume MFM division with a dedicated placental pathology service. Required milestones include formal credentialing in fetal Doppler ultrasonography, including supervised examinations spanning anatomy surveys, growth assessments, biophysical profiles, and the full Doppler spectrum; and a longitudinal case portfolio documenting fetal antecedents and neonatal outcomes for patients followed from MFM consultation through delivery. Additionally, there will be a 6-week placental pathology rotation with the Amsterdam classification as the certifying framework and a minimum of 30 placental-neonatal correlation reports, exposure to fetal therapy procedures (intrauterine transfusion, fetoscopic laser, in utero repair), and structured training in periviable counseling, integrating fetal prognosis and neonatal outcome data into a coherent family communication framework.

## Phase 2: neonatal intensive care (years 2–3)

Years 2 and 3 provide immersive Level IV NICU training identical in scope to a standard neonatology fellowship, with one structural addition: the Fetal-Neonatal Bridge Curriculum. For every patient in the trainee’s care, a bridge document is maintained that synthesizes prenatal history, Doppler and biophysical findings, placental pathology interpretation, effects of antenatal therapy, and a prospective admission-day prediction of expected morbidities. These predictions are reviewed weekly with a supervising fetoneonatologist, creating a continuous feedback loop between fetal prediction and neonatal reality that is entirely absent from current training. Procedural milestones include endotracheal intubation, umbilical line placement, thoracentesis, lumbar puncture, cranial ultrasound interpretation, echocardiographic PDA assessment, and aEEG interpretation.

## Phase 3: integration, research, and advanced practice (years 4–5)

Phase 3 is strongly recommended for trainees pursuing academic fetoneonatology positions and essential for those who will staff Fetoneonatology Continuity Services (described below). It is presented as an advanced track rather than a universal requirement in recognition of the current financial realities facing trainees, specifically the burden of medical school debt averaging $300,000 or more among U.S. graduates, and in acknowledgment that a mandatory 5-year fellowship would, at this stage of the discipline’s development, risk limiting interest to a prohibitively narrow pool. We believe, however, that Phase 3 represents the fullest expression of the specialty’s goals in the creation of a physician who provides genuinely longitudinal care across the fetal-neonatal continuum in real time. Programs are strongly encouraged to offer Phase 3 as the normative track, with the core 3-year fellowship serving as the minimum qualification for clinical practice.

The defining clinical structure of Phase 3 is the Fetoneonatology Continuity Service, in which trainees provide longitudinal care to a panel of high-risk pregnant women from MFM referral through NICU discharge, attending prenatal visits, managing deliveries, and following patients in the NICU as the primary physician under supervision. Research training culminates in an original contribution addressing a question within the fetal-neonatal continuum. A formal AI curriculum covers the principles of clinical machine learning, model performance evaluation, algorithmic bias, particularly regarding race and socioeconomic factors in perinatal outcomes [15], the regulatory framework for AI medical devices, and the ethics of algorithmic decision support in neonatal medicine.

## The fetoneonatologist in daily practice

Reviewer and reader alike may reasonably ask: what does a fetoneonatologist actually do on a Tuesday afternoon? The answer depends on the career track and institutional model, but the core clinical activities can be concretely described.

In the prenatal setting, the fetoneonatologist holds a joint prenatal consultation clinic alongside MFM, seeing high-risk pregnancies from approximately 22 to 34 weeks of gestation. Referrals come from MFM for fetuses with growth restriction, abnormal Doppler surveillance, multiple gestation complications, congenital anomalies amenable to postnatal management, and pregnancies complicated by conditions with strong fetal-neonatal pharmacologic implications (maternal diabetes, autoimmune disease, cardiac disease). In this clinic, the fetoneonatologist reviews fetal Doppler trajectories, interprets biophysical profiles, synthesizes antenatal therapy exposure histories, and communicates expected neonatal morbidity trajectories to families with a specificity that neither the MFM specialist nor the traditional neonatologist is fully equipped to provide. This prenatal clinic function is the fellowship’s most novel clinical contribution: a physician trained in both the fetal surveillance language and the neonatal outcome literature simultaneously present at the table.

At delivery, the fetoneonatologist attends high-risk births as a full clinical partner, integrating knowledge of the fetal surveillance history, placental biology, and antenatal therapy pharmacology into immediate delivery room management decisions. The fetoneonatologist who attended the prenatal clinic, reviewed the Doppler trajectory, and predicted polycythemia and mesenteric vulnerability in the growth-restricted 27-weeker arrives in the delivery room with a management plan, not a blank clinical slate.

In the NICU, the fetoneonatologist functions either as the attending of record on a dedicated Fetoneonatology Service, analogous to a subspecialty consultation service, or as an integrated member of the NICU team with specific responsibility for the fetal-neonatal bridge function: reviewing placental pathology reports within 72 h of delivery, updating management recommendations in light of pathology findings, and maintaining the bridge document that connects intrauterine exposures to postnatal clinical decisions. Whether the fetoneonatologist runs a dedicated service or functions as a NICU attending with a bridging role will depend on institutional volume, staffing, and program maturity. Both models are viable since the core function is the same.

Post-discharge, the fetoneonatologist may participate in neurodevelopmental follow-up clinics for NICU graduates, applying the DOHaD framework to longitudinal risk communication and connecting early-life intrauterine exposures to the developmental and cardiometabolic monitoring plan. This post-NICU continuity function, while not universally required, represents the logical downstream extension of the prenatal-to-NICU longitudinal model.

## Certification and implementation pathways

Formal establishment of fetoneonatology as an ACGME-accredited subspecialty is the long-term goal, but the pathway to that goal is best understood as a staged process. The regulatory burden for new ACGME subspecialty accreditation is substantial, and the evidence base required to satisfy it does not yet exist. The practical entry point is therefore the super-fellowship model, which does not require ACGME accreditation and can be initiated at individual academic centers under the umbrella of existing neonatology or MFM fellowship programs. Super-fellowships in neonatology have demonstrated that this model can generate both clinical excellence and outcomes evidence that eventually supports formal subspecialty recognition.

For the fetoneonatology super-fellowship, the immediate regulatory stakeholders are the ABP and individual academic institutions. The ABP would need to be willing to recognize fetoneonatology training as a distinct credential, either as a certificate of added qualification or, ultimately, as a subspecialty certification, and to develop or endorse an appropriate examination framework. The American Board of Obstetrics and Gynecology (ABOG) may become a relevant partner if the fellowship evolves toward joint ABP-ABOG certification for graduates of obstetric residencies. Professional society engagement, from the Society for Maternal-Fetal Medicine and the Section on Neonatal-Perinatal Medicine, will be important for advocacy, curriculum endorsement, and defining clinical privileges, but formal regulatory approval from these organizations is not required to launch the fellowship.

Pilot programs should be established at the ten to fifteen academic medical centers that currently maintain the required infrastructure, which includes a Level IV NICU, a high-volume MFM service, a dedicated placental pathology service, and a perinatal research program. Prospective outcome measurement should track neonatal morbidity rates in infants cared for by fetoneonatology-trained physicians, family communication quality scores, missed prenatal-neonatal correlations documented in bridge records, and trainee preparedness assessments. This evidence base, accumulated over 3 to 5 years of pilot experience, would then support a formal petition to the ACGME for subspecialty accreditation and to the ABP for certifying examination development.

## Objections and responses

### Will depth be sacrificed for breadth?

No. The neonatal component of fetoneonatology training is identical in duration and clinical exposure to current fellowship training. What changes is the interpretive framework. Neonatology taught with continuous reference to its fetal origins is not diluted neonatology; it is enriched neonatology. The fetal medicine competency is genuine clinical proficiency, credentialed in Doppler ultrasonography, not a survey course. The specialty is broader than its components, not shallower than either.

### Does this create new fragmentation?

The concern is misapplied. The fetoneonatologist does not replace the MFM specialist or the neonatologist. The fetoneonatologist provides the integrative function that currently does not exist. A single physician who translates prenatal findings into neonatal management decisions and provides continuity of reasoning and family communication across the delivery room. This reduces hand-offs at the most consequential transition in a patient’s early life.

### Where is the evidence of outcomes?

No randomized trial of fetoneonatology care versus standard care yet exists, and we do not claim otherwise. What exists is a large and convergent body of evidence that fragmented prenatal-neonatal care causes measurable harm, that the biological unity of the patient demands a corresponding clinical unity, and that the knowledge required to optimize outcomes is currently distributed across specialties in ways that guarantee knowledge gaps at the clinical interface. We would not accept the absence of a trial as justification for continuing a structurally defective drug delivery system. We should not accept it as justification for continuing a structurally defective care delivery system.

### Will declining interest in neonatology undermine recruitment?

This is a legitimate concern, as applications to neonatology fellowships in the United States have declined steadily, raising genuine questions about whether a new and more demanding subspecialty can attract sufficient interest. We offer three responses. First, broadening eligibility beyond pediatric residency to graduates of obstetrics and gynecology, maternal-fetal medicine, family medicine, and surgery, substantially expands the potential applicant pool and may attract physicians who find the integrated fetal-neonatal model more compelling than either discipline alone. Second, the emergence of a formally defined subspecialty with a distinct intellectual identity, novel clinical function, and clear career pathway may itself generate enthusiasm among trainees who perceive current neonatology training as insufficiently differentiated from what it was a generation ago. Third, the super-fellowship entry model does not require large-scale recruitment to succeed. Two to four fellows per program at a handful of pioneering academic centers is sufficient to generate the outcomes, evidence, and the proof of concept needed to build the field.

### Is a five-year fellowship financially practical given student debt burdens?

This is the most consequential practical objection, and it deserves a direct response. The average U.S. medical school graduate carries approximately $300,000 in student loan debt. A 5-year fellowship, even with stipend support, represents a substantial additional opportunity cost and delay in attending-level compensation. We take this concern seriously, and it is precisely why Phase 3 is structured as a strongly recommended but not universally mandatory track, with the core 3-year fellowship serving as the minimum qualification for practice.

For the financing of extended training, several mechanisms warrant exploration. Academic medical centers with existing NIH or institutional research infrastructure may be able to support Phase 3 through research training grants (T32, K08, K23), which provide stipend support independent of GME funding. GME funding through the Centers for Medicare and Medicaid Services typically supports up to 3 years of subspecialty fellowship training; institutions launching fetoneonatology super-fellowships would need to develop supplementary funding for Years 4 and 5 through departmental or philanthropy sources. Loan forgiveness programs, including the Public Service Loan Forgiveness program available to trainees at non-profit academic medical centers, can substantially mitigate the debt burden for those who pursue extended training. These solutions are not hypothetical: they are precisely the mechanisms that have supported the development of other long-advanced training programs in academic medicine, including cardiothoracic surgery, pediatric cardiac surgery, and complex MFM subspecialization.

The job market for fetoneonatology graduates, at least initially, will be concentrated in the academic medical centers with the infrastructure to support the specialty. This is not a limitation unique to fetoneonatology, but it is the standard profile of any new academic subspecialty in its early development phase. As the specialty matures and its outcomes evidence accumulates, the clinical model may diffuse to community Level III NICUs that serve high-risk perinatal populations, expanding the employment landscape. The prenatal consultation clinic model, which generates billable professional encounters, also provides a sustainable clinical revenue stream distinct from NICU attending income, a feature that strengthens the financial case for fetoneonatology positions within academic departments.

## Conclusions

The fetus and the neonate are one patient. This is a biological fact, established by decades of research in placental biology, developmental physiology, epigenetics, and the DOHaD. It is a fact that our training structures, certifying bodies, and clinical organizations have consistently failed to honor.

The fetoneonatology fellowship described is not a theoretical construct. The science that demands it is mature. The clinical problems it addresses are among the most consequential in medicine. The institutional infrastructure to pilot it exists at a handful of academic centers today. The regulatory pathway is clear: begin as a super-fellowship, build the outcomes evidence, and pursue formal ACGME accreditation and ABP certification in sequence. What is required is the recognition by program directors, professional societies, certifying boards, and the physicians who will build this field that the delivery room door has never been a biological boundary, and that it is time to stop treating it as one. Every high-risk fetus admitted to a Level IV NICU crosses that door without a physician trained to follow. The fetoneonatology fellowship is the proposal that one should.

## References

[1] Baschat AA, Cosmi E, Bilardo CM, Wolf H, Berg C, Rigano S, et al. Predictors of neonatal outcome in early-onset placental dysfunction. Obstet Gynecol. 2007;109:253–61. 17267821 10.1097/01.AOG.0000253215.79121.75

[2] Redline RW. Classification of placental lesions. Am J Obstet Gynecol. 2015;213:S21–28. 26428500 10.1016/j.ajog.2015.05.056

[3] Avery ME, Mead J. Surface properties in relation to atelectasis and hyaline membrane disease. Am J Dis Child. 1959;97:517–23. 10.1001/archpedi.1959.02070010519001 13649082

[4] Gantert M, Been JV, Gavilanes AW, Garnier Y, Zimmermann LJI, Kramer BW. Chorioamnionitis: a multiorgan disease of the fetus? J Perinatol. 2010;30:S21–30. 20877404 10.1038/jp.2010.96

[5] Ballabh P. Intraventricular hemorrhage in premature infants: mechanism of disease. Pediatr Res. 2010;67:1–8. 19816235 10.1203/PDR.0b013e3181c1b176 PMC2799187

[6] Neu J, Walker WA. Necrotizing enterocolitis. N Engl J Med. 2011;364:255–64. 21247316 10.1056/NEJMra1005408 PMC3628622

[7] Redline RW, O’Riordan MA. Placental lesions associated with cerebral palsy and neurologic impairment following term birth. Arch Pathol Lab Med. 2000;124:1785–91. 11100058 10.5858/2000-124-1785-PLAWCP

[8] Jobe AH, Bancalari E. Bronchopulmonary dysplasia. Am J Respir Crit Care Med. 2001;163:1723–9. 11401896 10.1164/ajrccm.163.7.2011060

[9] Bodenheimer T. Coordinating care — a perilous journey through the health care system. N Engl J Med. 2008;358:1064–71. 18322289 10.1056/NEJMhpr0706165

[10] Barker DJ. The fetal and infant origins of adult disease. BMJ. 1990;301:1111. 2252919 10.1136/bmj.301.6761.1111 PMC1664286

[11] Gluckman PD, Hanson MA, Cooper C, Thornburg KL. Effect of in utero and early-life conditions on adult health and disease. N Engl J Med. 2008;359:61–73. 18596274 10.1056/NEJMra0708473 PMC3923653

[12] Liggins GC, Howie RN. A controlled trial of antepartum glucocorticoid treatment for prevention of the respiratory distress syndrome in premature infants. Pediatrics. 1972;50:515–25. 4561295

[13] NIH Consensus Development Panel. Effect of corticosteroids for fetal maturation on perinatal outcomes. JAMA. 1995;273:413–8. 10.1001/jama.1995.03520290065031 7823388

[14] Romero R, Chaemsaithong P, Docheva N, Korzeniewski SJ, Tarca AL, Bhatti G, et al. Clinical chorioamnionitis at term IV: the maternal plasma cytokine profile. J Perinat Med. 2016;44:77–98. 10.1515/jpm-2015-0103 PMC5624710 26352068

[15] Topol EJ. High-performance medicine: the convergence of human and artificial intelligence. Nat Med. 2019;25:44–56. 10.1038/s41591-018-0300-7 30617339

